# Vertebral artery aneurysm rupture and hemothorax in a patient with neurofibromatosis Type-1: A case report and review of the literature

**DOI:** 10.1016/j.heliyon.2019.e02201

**Published:** 2019-08-01

**Authors:** Hael F. Abdulrazeq, Ira M. Goldstein, Samer T. Elsamna, Beth A. Pletcher

**Affiliations:** a Wayne State University School of Medicine, Department of Neurosurgery, Detroit, MI, USA; b Department of Neurological Surgery, Rutgers New Jersey Medical School, Newark, NJ, USA; c Division of Clinical Genetics, Department of Pediatrics, Rutgers New Jersey Medical School, Newark, NJ, USA

**Keywords:** Neurology, Neurofibromatosis, Vertebral artery aneurysm, Vasculopathy

## 1 Introduction

Neurofibromatosis type-1 (NF-1) is an autosomal dominant genetic condition caused by mutation in the neurofibromin gene. Classic symptoms include neurofibromas, hyperpigmented cutaneous spots (café-au-lait macules), axillary and/or inguinal freckling, iris hamartomas (Lisch nodules), and bone lesions [1]. Rarely, patients with NF-1 can have vascular abnormalities such as aneurysms, stenoses, and arteriovenous malformations. The exact pathogenesis of these abnormalities is unknown, but is thought to be due to alteration of neurofibromin expression in endothelial and smooth muscle cells of blood vessels [2]. Most patients remain asymptomatic. The most common site of involvement was reported to be the renal artery, which leads to renal artery hypertension [2].

We report a case of a 30-year-old female with NF-1 who presented with a spontaneous rupture of a vertebral artery (VA) aneurysm. A review of the literature by Oderich et al. found 46 reports of carotid, vertebral, and cerebral aneurysms, and noted that they occur most commonly in the third decade of life and are more frequent in women [3]. However, extracranial vertebral artery aneurysm in neurofibromatosis type 1 is very rare. Our review of the literature reveals 24 cases of extradural vertebral artery aneurysms in patients with NF-1, 12 of which ruptured and caused hemorrhage [4, 5, 6, 7, 8, 9, 10, 11, 12, 13, 14, 15, 16, 17, 18, 19, 20, 21, 22, 23, 24] (Table 1).

**Table 1:** Cases of Vertebral Artery Aneurysm in association with NF-1.

Case Number	Author/Year	Age (years)/Gender	Ruptured or Unruptured	Side/Level/AVF	Treatment	Symptoms	Outcome
1	Uneda et al. [4]/2016	35/F	Ruptured	R/C3–C4/AVF	Endovascular (coil)	Neck and shoulder pain	Recovery
2	Pentecost et al. [34]/1981	1/F	Unruptured	L/Th1/No	Observation	Limited range of motion, weakness	Disabled
3	Schubiger and Yasargil [24]/1978	50/M	Unruptured	L/C2–C6/No	Surgery	Radiculopathy	Recovery
4	Detwiler et al. [23]/1987	52/F	Unruptured	L/C2/No	Endovascular (balloon)	Neck mass, pain, bruits	Recovery
5	Negoro et al. [7]/1990	47/F	Ruptured	L/C1/No	Endovascular (balloon)	Neck pain, cervical hematoma	Recovery
6	Muhonen et al. [22]/1991	52/F	Unruptured	L/C2/No	Endovascular (balloon)	Neck mass and pain, arm weakness	Recovery
7	Schievink and Piepgras [21]/1991	43/F	Unruptured	L/C7/No	Observation	None	Recovery
8	Ohkata et al. [20]/1994	48/F	Unruptured	L/C4–C7/No	Surgery	Radiculopathy	Recovery
9	Horsley et al. [11]/1997	56/F	Ruptured	L/C5–C7/No	Endovascular (coil)	Neck pain and mass, arm parasthesias	Recovery
10	Hoffmann et al. [13]/1998	59/M	Unruptured	R/C6/No	Observation	None	Recovery
11	Ushikoshi et al. [19]/1999	40/F	Ruptured	L/C1/AVF (secondary)	Endovascular (balloon)	Occipitalgia, cervical hematoma	Recovery
12	Miyazaki et al. [9]/2004	52/F	Ruptured	L/C5–C7/No	Endovascular (balloon), surgery	hemothorax, radiculopathy, hypotension, altered mental status	Death
13	Arai et al. [17]/2007	38/M	Ruptured	L/-/No	None	Angina, dizziness, vomiting, hemothorax	Death
14	Hieda et al. [28]/2007	36/F	Ruptured	L/-/No	Endovascular (coil, n-butyl cyanoacrylate)	Back pain, angina, dyspnea, hypotension, hemothorax, coma	Death
15	Hiramatsu et al. [14]/2007	67/M	Unruptured	L/proximal vertebral artery/No	Endovascular (coil)	Dizziness	Recovery
16	Pereira et al. [18]/2007	14/F	Unruptured	R/C5–C6/No	Endovascular (balloon)	Radiculopathy	Recovery
17	Peyre et al. [6] 2007	18/F	Unruptured	R/C5–C6/No	Endovascular (coil)	Radiculopathy	Recovery
18	Horie et al. [12]/2008	30/F	Unruptured	R/C6–C7/No	Endovascular (coil, balloon)	Radiculopathy	Recovery
19	Higa et al. [27]/2010	60/F	Ruptured	L/-/No	Endovascular (coil)	Cervical hematoma, stridor, respiratory failure	Disabled
20	Morvan et al. [8]/2011	36/F	Ruptured	L/C3–C4/No	Endovascular (coil, stent)	Head and neck pain, vomiting, subarachnoid hemorrhage	–
21	Hiramatsu et al. [15]/2012	31/M	Ruptured	R/C6/No	Endovascular (coil)	Radiculopathy, neck pain, cervical hematoma	Recovery
22	Gouaillier-Vulcain F et al. [16]/2014	32/M	Unruptured	L/C8/No	Surgery, endovascular (stent)	Radiculopathy	Recovery
23	CY Lin et al. [10]/2017	18/F	Ruptured	L/proximal/No	Endovascular (coil, stent)	Seizures, neck swelling	Recovery

## 2 Discussion

This is a 30-year-old female with a past medical history of neurofibromatosis type 1, anterior cervical discectomy and fusion (ACDF) and solid posterior fusion of most of the cervical spine due to kyphosis, a large syrinx in the spinal cord, and hydrocephalus, status-post ventriculoperitoneal (VP) shunt in 2001, who presented to the emergency department (ED) with altered mental status. Per her family, the patient started complaining of headache and sudden onset right neck and chest pain, and subsequently fell to the ground. Since then, the patient experienced generalized pain and had become increasingly lethargic. She had cough and nasal congestion for the past week but had otherwise been well until the onset of these symptoms. The patient's history is notable for VP shunt malfunction in 2013. Upon arrival to the ED, the patient was found to be extremely hypotensive and hypothermic, with a blood pressure range of (65–95)/(37–60), and a temperature of 92.1 F (33.4 C). She was intubated in the ED, was unresponsive to 4 L of IV fluids, and was started on pressors for the hypotension. On physical exam, the patient was lethargic and in significant distress secondary to pain. She opened her eyes to pain, followed commands, and moved all extremities purposefully. Her pupils were equal, round, and reactive to light, and extraocular movements were intact. Severe nuchal rigidity was noted. Her shunt depressed and refilled on examination. Abdomen was soft and nontender. Labs were obtained, and her electrolytes were found to be within normal limits, hemoglobin was 11.2 g/dL, and white blood count was 12.1. Her partial thromboplastin time (PTT) and international normalized ratio (INR) were normal at 30.0 and 1.16, respectively.

**Fig. 1:**
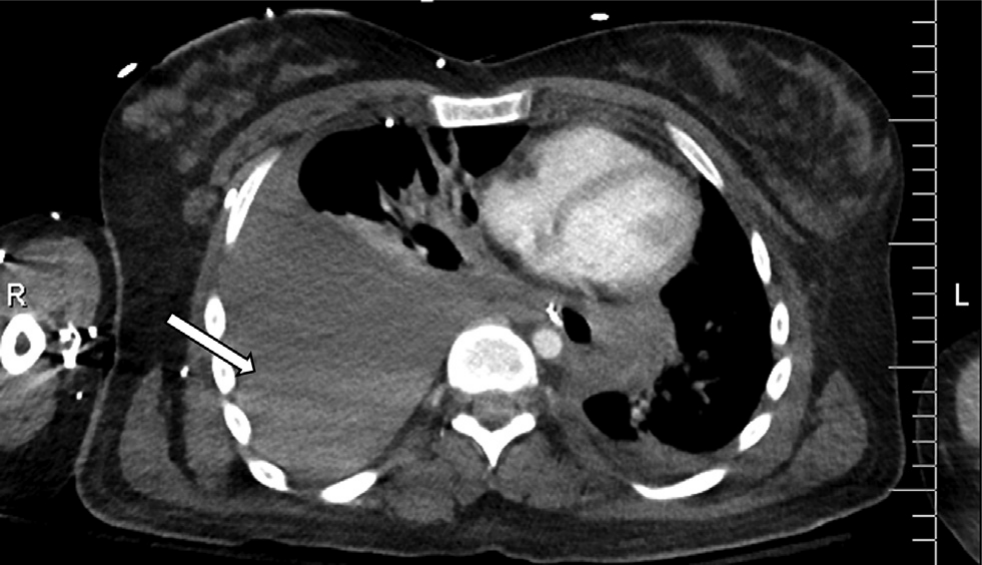
CT of chest showing large right pleural effusion with layering hyperdensity compatible with hemothorax.

**Fig. 2:**
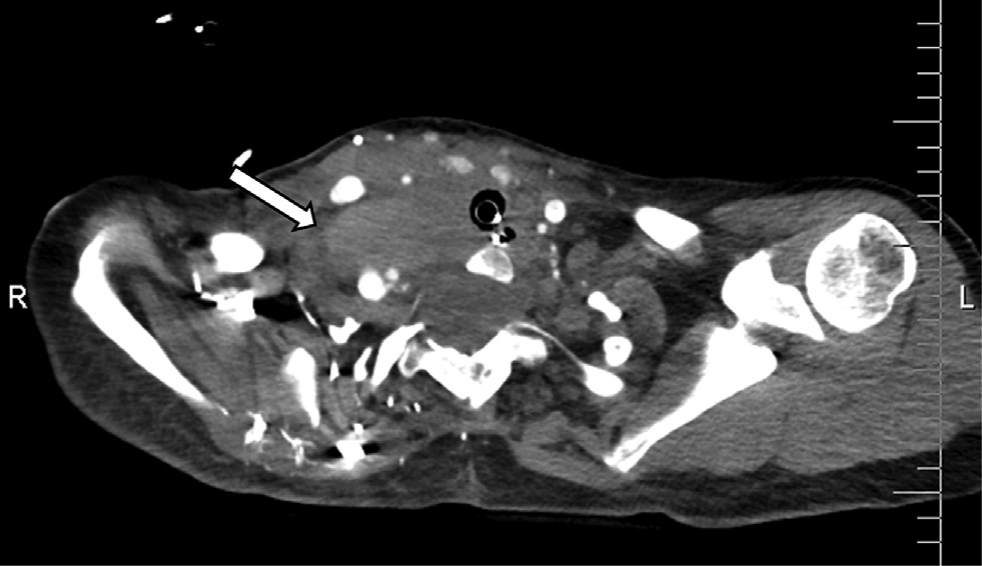
CT soft tissue neck with contrast demonstrating soft tissue prominence in the right supraclavicular region with a blush of contrast posteriorly suggestive of evolving hematoma with arterial extravasation.

**Fig. 3:**
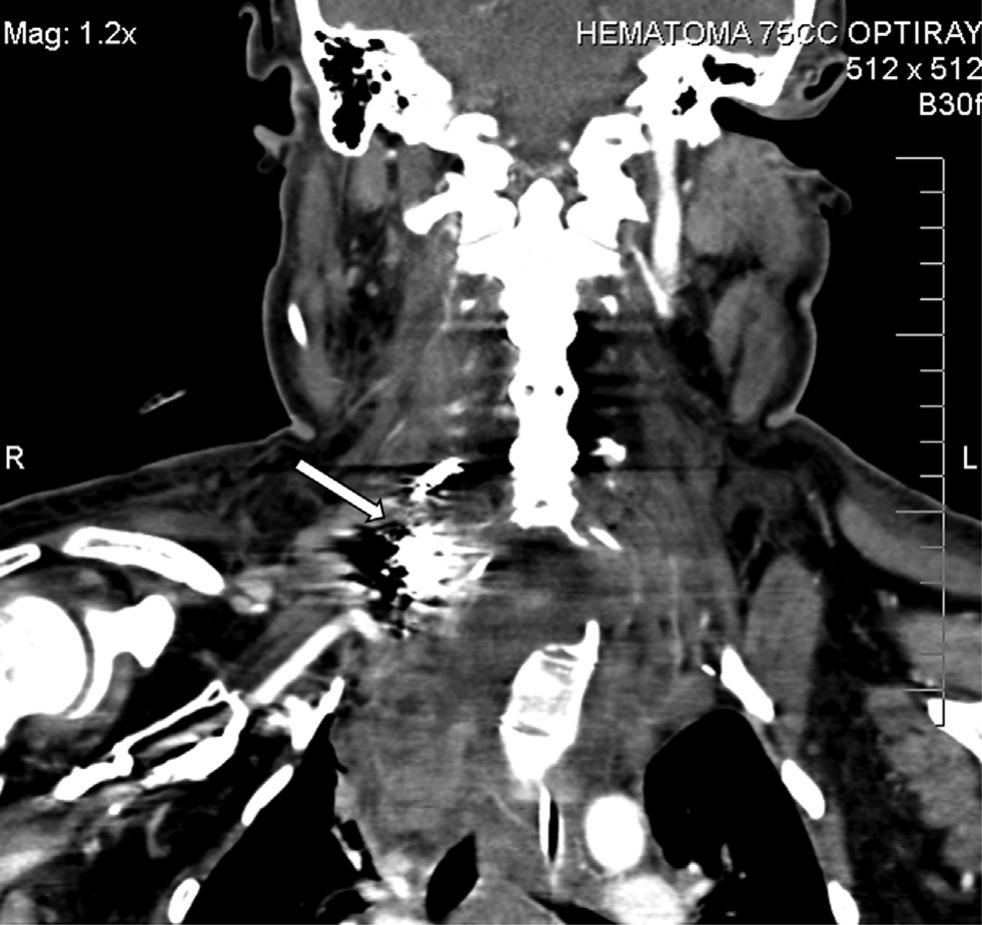
CT Neck status post coiling and hematoma evacuation, revealing embolic coil material in the proximal right vertebral artery. Hematoma decreased in size from Fig. 1 with decrease in degree of tracheal deviation.

The patient had a computerized tomography (CT) study which revealed a right vertebral artery (VA) aneurysm and right hemothorax (Fig. 1). A chest tube was placed. CT soft tissue of the neck with contrast showed soft tissue prominence in the right supraclavicular region with a blush of contrast posteriorly suggestive of evolving hematoma with arterial extravasation (Fig. 2). There were some delays in the proper assessment of the patient's status and severity of their condition, possibly due to lack of effective communication. Patient was eventually taken for a cerebral angiogram on hospital day two. Coil embolization was performed of the right VA/V1 segment pseudoaneurysm with therapeutic right VA sacrifice (Fig. 3). Patient received 2 units of packed red blood cells intraoperatively. The patient continued to have symptoms of airway obstruction and was taken to the operating room on hospital day seven to address the right neck hematoma and facilitate intra-operative extubation. The procedure revealed copious amounts of blood with large clots which were evacuated from the right posterior lateral neck and supraclavicular region that had been causing tracheal deviation to the left. Extubation was successful, and a surgical drain was placed in the right paramedian supraclavicular region. The patient was then transferred to the surgical intensive care unit for medical management. The patient was discharged home after stabilization and was back to baseline. She followed up at clinic for the subsequent months. Unfortunately, she died of an unrelated accidental traumatic brain injury shortly after her hospitalization.

## 3 Conclusions

NF-1, a well-described neurocutaneous condition, is known for the ubiquitous effects on the nervous system as well as other organ systems and renders patients more susceptible to numerous complications such as tumors and vascular pathologies [25]. This is similar to the increased risk for developing cerebrovascular diseases notable in patients with certain connective tissue disorders such as Marfan syndrome, Ehler-Danlos sydrome, and Loeys-Dietz syndrome [26]. These disorders, in addition to NF-1, are linked to vasculopathies including arterial aneurysms because of their involvement of components of the extracellular matrix, which compromises the integrity of vessel walls.

Routine screening for these vasculopathies is not recommended, due to their rare incidence. When aneurysms are discovered incidentally on imaging, a decision must be made to pursue operative treatment versus conservative management. Large aneurysms are more likely to undergo open reconstruction, as endovascular treatment has not yet been standardized for aortic or renovascular aneurysms [3]. Lesions affecting the abdominal aorta or the renal artery causing claudication or renovascular hypertension, respectively, may prompt operative intervention.

Vertebral artery aneurysms can present with various symptoms and findings [3], including neck pain; neck mass; respiratory compromise due to mass effect of aneurysms or hematomas; upper extremity neurologic symptoms due to compression of the brachial plexus; neurologic symptoms related to the VA territory; and bony erosion of the vertebrae [3].

Vertebral artery aneurysm rupture can be a fatal condition if not recognized promptly and managed aggressively, especially when the patient presents in hemorrhagic shock. Management options include surgical or endovascular treatment, or observation. Surgical treatment carries a higher risk of exsanguination, and observation in many cases results in death [17, 27]. Operative treatment with endovascular coiling is necessary for prevention of further complication and potentially fatal hemorrhage, as this method of treatment in minimally invasive and shorter in duration. Very rarely, the aneurysm can hemorrhage into the thoracic cavity, leading to hemothorax [28]. Hemorrhagic shock due to hemothorax in NF-1 patients can alternatively be caused by hemorrhagic mediastinal tumors or erosion of thoracic vessels by a tumor [29, 30, 31], but when an aneurysm is identified in patients with hemothorax, intervention becomes necessary after hemodynamic stabilization [29]. It is important to note that a variety of aneurysms have been reported with different arterial origins causing intrathoracic hemorrhage, and various arteries have been identified as sources of bleeding, such as intercostal and subclavian arteries, often secondary to enlarging tumors [29, 32].

In patients with airway compromise, early evacuation of resultant hematomas in the laterocervical compartment and surgical drainage is necessary, which can also be seen in the case report by Bissacco et al. [33] As such, extra caution should be taken when a patient with NF-1 presents acutely with complaints of neck pain and/or symptoms of hemorrhage or shock [3, 34]. In the cases of patients who are diagnosed with an aneurysm that has not ruptured yet, it critical to serially monitor these vessels and assess for progression and intervene as needed [35].

## Declarations

### Author contribution statement

All authors listed have significantly contributed to the investigation, development and writing of this article.

### Funding statement

This research did not receive any specific grant from funding agencies in the public, commercial, or not-for-profit sectors.

### Competing interest statement

The authors declare no conflict of interest.

### Additional information

No additional information is available for this paper.
